# Water-in-Oil Nano-Emulsions Prepared by Spontaneous Emulsification: New Insights on the Formulation Process

**DOI:** 10.3390/pharmaceutics13071030

**Published:** 2021-07-07

**Authors:** Salman Akram, Nicolas Anton, Ziad Omran, Thierry Vandamme

**Affiliations:** 1 Faculty of Pharmacy, Université de Strasbourg, CNRS, CAMB UMR 7199, F-67000 Strasbourg, France; salman.akram16@gmail.com (S.A.); nanton@unistra.fr (N.A.); 2 INSERM, Regenerative Nanomedicine UMR 1260, Centre de Recherche en Biomédecine de Strasbourg (CRBS), Université de Strasbourg, F-67000 Strasbourg, France; 3 Pharmacy Program, Department of Pharmaceutical Sciences, Batterjee Medical College, Jeddah 21442, Saudi Arabia

**Keywords:** nano-emulsion, water-in-oil, spontaneous emulsification, low-energy method, surfactant

## Abstract

Nano-emulsions consist of stable suspensions of nano-scaled droplets that have huge loading capacities and are formulated with safe compounds. For these reasons, a large number of studies have described the potential uses of nano-emulsions, focusing on various aspects such as formulation processes, loading capabilities, and surface modifications. These studies typically concern direct nano-emulsions (i.e., oil-in-water), whereas studies on reverse nano-emulsions (i.e., water-in-oil) remain anecdotal. However, reverse nano-emulsion technology is very promising (e.g., as an alternative to liposome technology) for the development of drug delivery systems that encapsulate hydrophilic compounds within double droplets. The spontaneous emulsification process has the added advantages of optimization of the energetic yield, potential for industrial scale-up, improved loading capabilities, and preservation of fragile compounds targeted for encapsulation. In this study, we propose a detailed investigation of the processes and formulation parameters involved in the spontaneous nano-emulsification that produces water-in-oil nano-emulsions. The following details were addressed: (i) the order of mixing of the different compounds (method A and method B), (ii) mixing rates, (iii) amount of surfactants, (iv) type and mixture of surfactants, (v) amount of dispersed phase, and (vi) influence of the nature of the oil. The results emphasized the effects of the formulation parameters (e.g., the volume fraction of the dispersed phase, nature or concentration of surfactant, or nature of the oil) on the nature and properties of the nano-emulsions formed.

## 1. Introduction

Nano-medicine has emerged in the past few decades as an important field of research due to its advantages over conventional therapeutic options [1,2]. Nano-emulsions (NEs) are one of the most important drug vehicles in nano-medicine, as they offer the possibility of high-efficiency delivery of lipophilic drugs loaded within the oily core of the droplets [3]. Anecdotally, hydrophilic molecules can also be encapsulated in water-in-oil NEs by direct entrapment in the oil nano-droplets [4,5,6], or by the use of complex double structures such as nano double emulsions [7,8,9]. The choices of oil and type and grade of surfactant, with a focus on the intended administration route, allow the production of NEs that are nontoxic and nonirritating, with improved drug bioavailability [10,11]. However, these choices can also determine many other NE properties, and therefore, their applications, including the NE physical stability and loading properties [12,13,14,15,16].

One important consideration is that even when NEs are thermodynamically unstable, the droplet suspension is kinetically stable, regardless of the sense of the emulsion, i.e., direct or reverse [17]. The Gibbs free energy ΔG of this type of droplet suspension is theoretically very high, since it relies on the interface increase ΔA, which is as high as the droplet size is small [18]. However, although the ΔG of a NE is much greater than that of a microscale emulsion, the NEs, in practice, appear significantly more stable or are “kinetically stable” for steric stabilization reasons [18,19]. Conversely, gravitational separation, sedimentation or creaming, is negligible with these Brownian droplets. In addition, Ostwald ripening induces only a small evolution of NEs, making the suspension highly stable over months [20,21,22].

NEs can be formed in several ways, classified mainly as high-energy and low-energy methods [19,23]. The most popular high-energy methods are high-pressure homogenization, micro-fluidization, and ultrasonication [18,19], whereas the low-energy methods, also known as transitional nano-emulsification methods [24,25], are mainly described as phase-inversion temperature methods and spontaneous emulsification. The advantages of low-energy over high-energy methods lie in the gain in energy yields, which is beneficial for industrial scale-up as well as in the fact that a low-energy process is itself soft, and prevents the potential denaturation or destruction of fragile molecules like proteins and peptides [26].

Our overall research objective is to encapsulate hydrophilic therapeutic agents in lipid droplets as a strategy for increasing their bioavailability after oral administration or for fabricating preparations for topical application. In the present study, our focus was on the formulation of water-in-oil (w/o) NEs by low-energy methods. As a preamble to the formulation of double emulsions (i.e., w/o NEs dispersed in an aqueous phase), a better understanding and optimization of the formulation of w/o low-energy NEs is provided. Spontaneous nano-emulsification methods and processes are well documented regarding oil-in-water (o/w) NEs, whereas the literature reports on the formulation of reverse w/o NEs by spontaneous methods still remain anecdotal, despite the great potential of these NEs.

A recent report [27] has provided a general description of this spontaneous w/o emulsification process, also called the emulsion inversion point (EIP), and showed that surfactant interactions with the phases strongly affect the emulsification. The efficiency of the formulation of w/o or o/w NEs was shown to depend on (i) the properties of the surfactants used (i.e., the hydrophilic-lipophilic balance [HLB] of the surfactant mixture), and (ii) the order of mixing of the different phases. Two methods were proposed and compared: in this reference, method A, where oil is slowly poured into the (water + surfactant) mixture, and method B, where water is slowly poured into the (oil + surfactant) mixture. Interestingly, for the formulation of o/w NEs, using a mixture of Tween 80 and Span 80 in different amounts to change the HLBs, methods A and B were roughly comparable and showed only a slight variation for specific HLB values. By contrast, the two methods showed clear differences in the formulation of w/o NEs, as method B only worked for lower HLBs, whereas method A was successful for a large range of HLB values. However, this previous study [27] focused primarily on investigations into o/w NEs and only showed the feasibility of formulating reverse w/o NEs.

In the present study, our objective was to conduct a more in-depth investigation of the spontaneous formulation process of NEs as a preliminary step for the fabrication of complex carriers for hydrophilic or both hydrophilic and lipophilic active ingredients. Based on the pioneering study discussed above [27], we proposed to compare method A and method B, but with a focus on w/o NEs and a deeper examination of the formulation and process parameters. We studied the impact of the process parameters, e.g., flow rate of the dropping phase and mixing rate of the suspension, as well as the impact of the phase ratio, surfactant ratio, surfactant mixture composition, and nature of the oils for both methods. Our aim in taking this approach was to identify the factors and the experimental conditions that substantially affect the process, as a way to clarify how to optimize the spontaneous w/o nano-emulsification. No literature with this focus has been published to date, making the present study an important step forward as a starting point for future innovations.

## 2. Materials and Methodology

### 2.1. Materials

The model oil chosen for the experiments was Labrafac^®^ WL 1349 (Gattefossé S.A., Saint-Priest, France). This oil is a mixture of capric acid and caprylic acid and is compatible with parenteral administration. Peanut oil, mineral oil, olive oil, soy bean oil, sesame oil, Span 80, and Span 85 were purchased from Sigma–Aldrich (Saint-Louis, MO, USA.). Polyglycerol polyricinoleate (PGPR) was kindly gifted by Stéarinerie Dubois (Boulogne-Billancourt, France). PGPR is an emulsifier largely used for human consumption, and is approved for use in food formulations by the Food and Drug Administration (FDA) and the Joint FAO/WHO Expert Committee on Food Additives (JECFA).

### 2.2. Methodology

**Figure 1:**
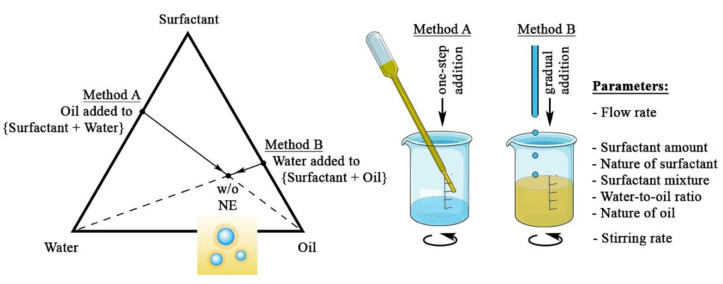
Schematic representation of the formulation pathways followed and compared in this study.

As presented in the introduction section, w/o NEs were made by two unique methods, method A and method B, as follows:

In method A, NEs were prepared by spontaneous emulsification involving a sudden mixing of the oil phase in an aqueous phase (water + surfactant) mixture (see Figure 1). The experiments were conducted in a 4 mL glass vial at room temperature. Initially, the water phase was prepared by mixing distilled water and lipophilic surfactants, followed by gentle magnetic stirring (500 rpm) for 5 min. The oil was then added instantaneously from a disposable pipette, and the mixture was homogenized by vortexing for 5 min. All experiments were conducted to ensure a consistent final NE volume of 2 mL.

In method B, NEs were prepared by spontaneous emulsification, but by gradually dropping the water phase into the (oil + surfactant) mixture, as illustrated in Figure 1. The experiments were conducted in a 100 mL beaker at room temperature. Initially, an oily phase was prepared by mixing the selected oil with lipophilic surfactants, pure or surfactant mixture, followed by gentle magnetic stirring for 5 min. Distilled water was then added to this mixture at a controlled rate using a peristaltic pump (Minipuls MP3 Drive, Gilson, France) over a predetermined time, with the rate and times defined by the experiment. All experiments were conducted to ensure a consistent final NE volume of 10 mL. Finally, the w/o NEs were subjected to further magnetic stirring for another 5 min, noting that further mixing would not change the size distribution of the NEs.

Method A and method B differed significantly in protocol, i.e., either a sudden addition of oil or gradual pouring of water, respectively, as the continuous phase of the generated NEs was the oil phase. That is to say, for method A, performing a gradual addition of oil would have an unfavorable effect on the emulsification and would potentially result in the initial formation of oil droplets in water due to the imbalanced volume fraction, followed by a catastrophic phase inversion upon further addition of oil. It means that the emulsion would be o/w, with the droplet’s size being much higher than the nanoscale. Therefore, the sudden addition of the whole oil phase would immediately induce a phase inversion and optimize the w/o nano-emulsification process. By contrast, for method B, the added water is immediately in a favorable configuration for spontaneous emulsification, since the continuous phase is oil, and the water penetrating the oil/surfactant phase rapidly breaks up into nano-droplets. In comparison with method A, the method B allowed a more complex investigation of the protocol by varying the flow or stirring rates.

The two methods are schematically represented in Figure 1 as a ternary diagram showing the pathways followed by the system up to the composition of the final NEs, noted as “w/o NE”. In principle, for final w/o NEs with an identical composition, choosing method A or method B, or modifying some process parameter, will modify the size distribution. Of course, changing the formulation parameters, such as the surfactant concentration or the water-to-oil ratio, will also modify the location of the w/o NE in the ternary diagram, as well as potentially change the size distribution. The figure summarizes all the studied parameters: flow rate, stirring rate, surfactant amount through the surfactant-to-oil weight ratio, $\mathrm{SOR}=\left(w_{surf.}\times 100\right)/\left(w_{surf.}+w_{oil}\right)$ (where $w_{surf.}$ and $w_{oil}$ are the weight of surfactant and oil, respectively), the nature and mixture of surfactants, the proportions of water and oil, through the water-to-oil weight ratio (WOR), and the nature of the oil.

### 2.3. Dynamic Light Scattering

The droplet size distribution, mean size, and polydispersity indexes (PDIs) were determined by dynamic light scattering (DLS) using a Malvern ZS90 instrument (Malvern, Orsay, France). The viscosity of the oil used, equal to the emulsion viscosity after dilution, was 30.0 mPa.s, and refractive indexes of oil and water were 1.446 and 1.33, respectively. Notably, the visual aspect of the reverse w/o NEs differed from the direct o/w NEs. In general, the diluted o/w NEs displayed a bluish aspect that aided the formulator in choosing the right dilution for the DLS measurements. Conversely, in the case of the w/o NEs, as we observed in the present study, the concentrated NEs presented a white and milky aspect, due to multiple diffusions of light, and, at a certain dilution with the same oil continuous phase (i.e., Labrafac^®^ WL 1349), the NE sample became fully transparent without any bluish aspect. Thus, we ensured that the DLS characterizations were correct by performing the following for each sample: (i) a dilution with oil, up to the transition to a transparent aspect and (ii) the addition of a small drop of NEs to set the sample at the frontier between these two aspects, as this corresponded to the optimal aspect for DLS measurements. It is interesting to note that the dilution of droplets in oil has no impact on their size and stability, for thermodynamic reasons. Indeed, once formed, the nano-droplets can be neither fractionated (size decrease) nor merge (size increase) upon a dilution with the continuous phase again. Each formulation test was repeated three times.

## 3. Results and Discussion

### 3.1. Impact of the Process Parameters

**Figure 2:**
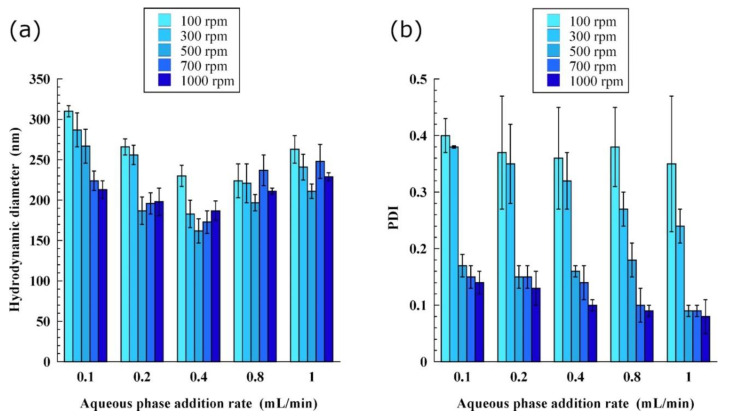
Influence of the flow rate of water addition and the stirring rate in beaker as process parameters that modify the nano-emulsion size (experimental conditions: method B, SOR = 20%, WOR = 20/80; the oil phase is a medium chain triglyceride (Labrafac^®^ WL 1349) and the surfactant is polyglycerol polyricinoleate (PGPR)): (**a**) Hydrodynamic diameter, and (**b**) polydispersity indexes (PDIs).

As discussed above, in method A, the whole oil phase is immediately added to the aqueous phase to induce the nano-emulsification. By contrast, in method B, the impact of the rate of water addition and stirring rate on the NE properties can be investigated to determine their incidence and to optimize the protocol. These results are summarized in Figure 2, with a representative case (SOR = 20%, WOR = 20/80; the oil phase is Labrafac^®^ WL 1349 and the surfactant is PGPR). In Figure 2a, at lower stirring rate, the size decreases with increases in the stirring rate. A combination of both stirring rate and water addition rate parameters, around 500 rpm and 0.4 mL/min, is needed to produce the smallest NEs. In parallel, the values of PDI shown in Figure 2b reveal the real importance of these parameters in the sample homogenization in the spontaneous process. A satisfactory dispersion parameter (PDI < 0.2) is obtained, at least for a stirring rate of 500 rpm, and this can further be increased (PDI < 0.1) with an increase in the stirring rate.

Interestingly, these two parameters are not usually thought to have a significant impact on the resulting NE size and size distribution [19]. However, we have shown that altering these process parameters can cause size differences of up to 100 nm. Increasing the mixing rate is generally reported to affect only the time necessary to stabilize and homogenize the sample [19]. Compared to high-energy processes, increasing the pressure, power, or other parameters favoring homogenization always helps to speed up the process and can favor droplet fractionation, thereby decreasing the droplet size. By contrast, further increases in these parameters can have the opposite effect, and induce a recombination and coalescence of the newly formed droplets before the surfactants have time to stabilize the interface [28,29]. Our procedure showed this type of trend, leading to the assumption that it is probable that the mixing quality impacts on the water phase fractionation into nano-droplets. Newly formed droplets have a greater tendency to re-coalesce at high stirring speeds and/or with increasing the addition rate of the dispersed phase.

### 3.2. Impacts of the Surfactant-to-Oil Ratio, the Nature of Surfactants, and the Surfactant Mixture

**Figure 3:**
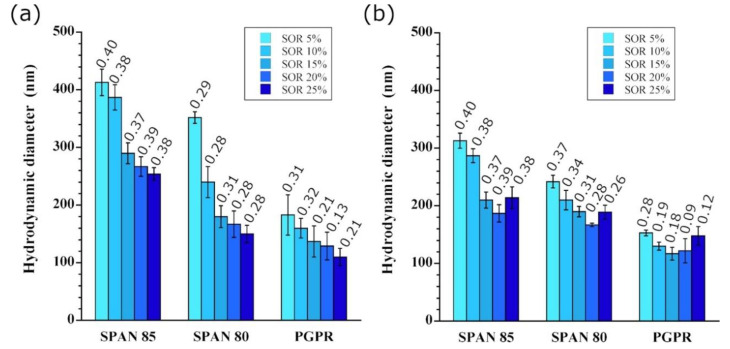
Influence of the nature of the surfactant (Span 85, Span 80, and polyglycerol polyricinoleate (PGPR)) and of the surfactant-to-oil ratio (SOR) on the efficiency of nano-emulsification, for (**a**) method A and (**b**) method B (water-to-oil ratio (WOR), stirring rate, and water addition rate for method B equal to 10/90, 500 rpm, and 0.4 mL/min, respectively; the oil is Labrafac^®^ WL 1349). Each value is labeled with the corresponding PDI.

The influences of the type of surfactant and surfactant to oil ratios were also compared for method A and method B. The WOR was kept constant at 10/90, while the SOR was varied from 5% to 25%, and three different surfactants (Span 85, Span 80, and PGPR) were compared. For method B, the stirring rate and water addition rate were defined as 500 rpm and 0.4 mL/min, respectively. The results are reported in Figure 3 and reveal some important information.

In general, increasing the surfactant amount results in a decrease in the droplet size, which is a general tendency for spontaneous nano-emulsification of direct o/w emulsions [30]. The various surfactants show clear differences, which were potentially expected, since the spontaneous emulsification mechanisms are based on the surfactant behavior. This explains the slight difference seen between method A and method B for Span 85 and Span 80, the results being similar for PGPR.

The first notable feature is the success of the spontaneous emulsification method in producing nano-droplets in a very simple fashion. However, the PDI values seem to indicate a suboptimal state of dispersion of the produced droplets (0.20 < PDI < 0.30) at most points, whereas the dispersion improves (PDI < 0.20) with the use of PGPR and SOR > 15%. As shown in Figure 2, further optimization is also possible with method B by increasing the stirring rate during emulsification leading to PDI ≤ 0.1.

Regarding method A, the sudden addition of oil to a phase containing a lipophilic surfactant likely induces a turbulent mechanism similar to the one described for direct nano-emulsification [30], as follows: (i) once the two phases come in contact, the surfactant is solubilized by the oil phase by a very fast penetration into the aqueous phase; (ii) a phenomenon based on the spinodal decomposition of the aqueous phase then occurs, causing fractionation at the nano-scale; (iii) the aqueous nano-droplets are ultimately formed and stabilized by the surfactants surrounding these droplets. According to method A, the emulsification is related to a sudden process governed by the interactions of the surfactants with the phases, as described for direct emulsions [27].

Regarding method B, the emulsification mechanism appears to be quite different. The oily phase that receives the dropping water phase is already a continuous phase, and the nano-emulsification begins with the first water drop. A possible mechanism could be the formation of microemulsions as swollen micelles, followed by equilibration between microemulsion domains and emulsion droplets. That is to say: (i) prior to water addition, the surfactant concentration in oil is sufficiently high enough to promote the formation of reverse micelles, as previously reported [5,6]; (ii) adding water to this type of phase then promotes the formation of a microemulsion described as a swollen micelle phase [31] that is able to adsorb a significant amount of water, depending on the surfactant concentration; (iii) further addition of water beyond the capacity of the microemulsion will create emulsion droplets that co-exist with the swollen micelles under the dynamic conditions of stirring and the ultra-low interfacial tension of the microemulsion, and this type of system is assumed to equilibrate toward the w/o NEs that are finally observed. A mechanism that would explain the observed effect of the surfactant concentration could be a conditioning of the spontaneous emulsification process by the NE self-assembly and micelle properties.

This point of view that correlates the spontaneous emulsification of water with the presence of reverse micelles in oil has recently been investigated with PGPR and medium-chain triglycerides [32], similar to the present study. The authors showed that the spontaneous formation of aqueous droplets in an oil phase does not originate due to pure interfacial tension or to a turbulence effect; rather, it depends on the PGPR concentration and the presence of micelles. These authors proposed that the forming droplets coarsen with time, thereby increasing in size and creating a system state that is thermodynamically unstable beyond the microemulsion of swollen micelles and promoting the formation of actual emulsion droplets. According to this process (method B), the comparison of Span 85, Span 80, and PGPR in Figure 3b reveals that this interpretation can be extended to various low-HLB surfactants and is possibly correlated with the value of their critical micelle concentrations (*cmc*) in oil. The ranges of *cmc* for these molecules provided in the literature, depending on the nature of the oil, are 4.7 to 6.0 wt.% for Span 85 [33], 1.2 to 3.0 wt.% for Span 80 [33,34], and 1.0 to 1.8 wt.% for PGPR [32,35]. Indeed, at similar surfactant concentrations, a lower *cmc* increases the concentration of the micelles. Thus, Figure 3b shows that increasing the concentration of micelles, by changing the nature and concentration of the surfactant, decreases the droplet size. This study emphasized a link between (i) the emulsification process (method B), (ii) the surfactant concentration, and (iii) the resulting droplet size. Increasing the surfactant amount, and thus the number of micelles, results in decreasing the droplets size. It follows that a higher shearing improves the homogeneity of the water spreading into micellar oil, and thus decrease the droplet’s size.

### 3.3. Impact of the Surfactant Mixture

**Figure 4:**
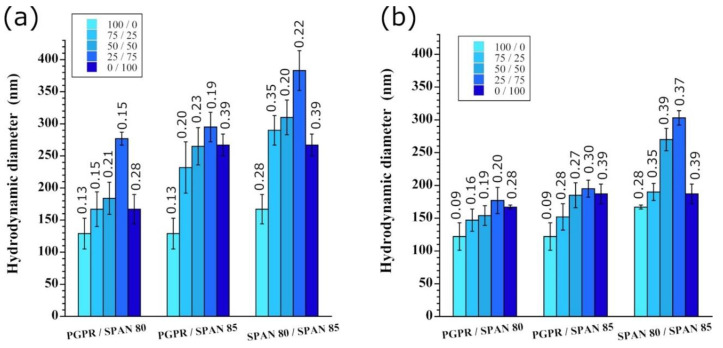
Influence of the mixture of surfactants (Span 85, Span 80, and polyglycerol polyricinoleate (PGPR)) on the efficiency of nano-emulsification, for (**a**) method A and (**b**) method B, and at the different weight ratios indicated as insets (SOR, WOR, stirring rate, and water addition rates for method B are fixed at 20%, 10/90, 500 rpm, and 0.4 mL/min, respectively; the oil is Labrafac^®^ WL 1349). Each value is labeled with the corresponding PDI.

The effect of the surfactant on the emulsification was also examined using a binary mixture of the surfactants studied above (at a fixed SOR of 20%, and WOR equal to 10/90). Figure 4a,b show a comparison of methods A and B, respectively. The mixture of surfactants gives larger droplet sizes compared to the pure surfactants, regardless of the method and the type of surfactant. The PDI values of the surfactant mixtures lie between those of the pure formulations, with optimal results given by PGPR or PGPR/Span 80.

With classical emulsification methods, a mixture of surfactants generally helps modify the *HLB_m_* (the HLB of the surfactant mixture) to fit the *HLB_R_* (the required HLB) of the oil to optimize the stability and efficiency of emulsification. This is due to a synergetic effect at the interface of the different surfactants, thereby allowing an optimized affinity with the phases compared to pure surfactants. Thus, the surfactants only play their common role as interfacial stabilizers. By contrast, in the present study, the spontaneous emulsification takes advantage of the different affinities and solubilities between the phases to create turbulences (method A) or to slightly perturb the properties of the micellar phases (method B) by the surfactant mixtures. These effects probably explain the obtained results.

### 3.4. Impact of the Fraction of the Disperse Aqueous Phase

**Figure 5:**
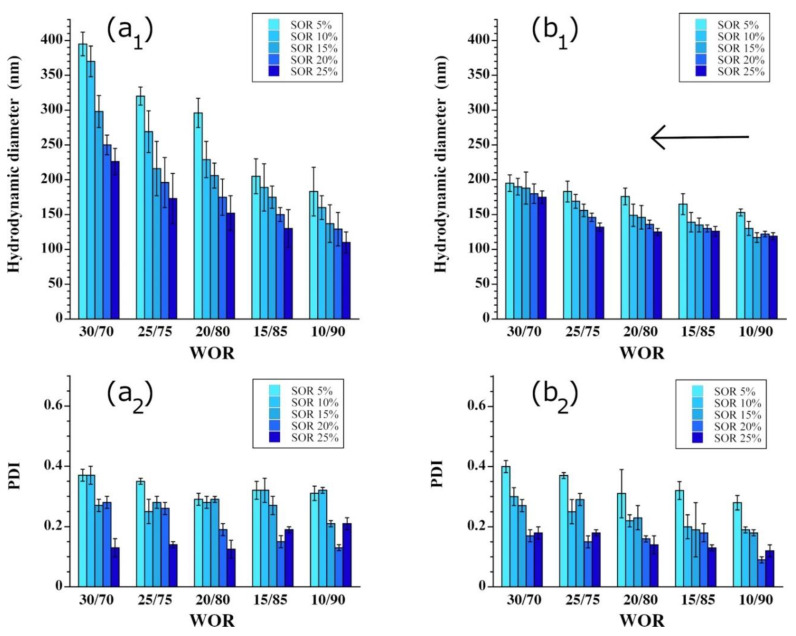
Study of the effects of the water-oil ratio (WOR) on the spontaneous emulsification process for (**a_1_**,**a_2_**) method A and (**b_1_**,**b_2_**) method B, and for different surfactant contents (surfactant-to-oil-weight ratio, SOR), as noted in the insets: The oil phase is a medium chain triglyceride (Labrafac^®^ WL 1349), the surfactant is polyglycerol polyricinoleate (PGPR), and the stirring rate and water addition rate for method B are fixed at 500 rpm and 0.4 mL/min, respectively. Subscript 1 shows the mean droplet hydrodynamic diameter, and Subscript 2 indicates the corresponding values of the polydispersity indexes (PDIs).

In addition to the role of surfactants, we also examined the role of the amount of disperse aqueous phase on the emulsification mechanisms. Figure 5 shows the effect of WOR values on different SOR values. The first observation is that the method choice clearly affects the size range of the droplets. In particular, method A (Figure 5a) seems to be significantly less efficient than method B (Figure 5b) for a WOR from 20/80 to 30/70, as it creates a much larger size and higher PDI values. The same trend is seen in the quality of the dispersion; for example, an SOR of 20% gives a PDI < 0.2 for WOR = 30/70 with method B, whereas this PDI is obtained at WOR = 20/80 with method A.

Interestingly, this series of experiments definitively shows the importance of the experimental procedure and the choice of the spontaneous method. For a strictly identical composition, this difference in the w/o NE properties arises fundamentally from the physicochemical behaviors of the three compounds and their respective interactions in response to the order of mixing. Since method A is apparently governed by a catastrophic phase inversion, along with a transfer of amphiphiles from the aqueous phase to the oil, NE formation is a sudden one-step process. Thus, by definition, it is less controllable than the formation process that governs method B. In method B, the dispersed aqueous phase has time to equilibrate with the reverse micellar phase before further water is added, and the phase can equilibrate with the former state to gradually give rise to a more homogeneous dispersion. Overall, this observed difference between method A and method B is confirmed by the different results of this study, and should probably be considered a rule for this type of emulsification process.

More specifically, for method B, the addition of water to the (oil + surfactant) phase is gradual (0.4 mL/min); therefore, the whole system composition changes during the process, as indicated by the arrow in Figure 5b_1_. As the water addition rate is constant, changing the WOR of the system, in this process, only depends on time. Then, we can note that the system passes through the different WOR before the selected value (e.g., 10/90, then 20/80, then 30/70, etc.) as indicated with the arrow in Figure 5b_1_. We can also notice that the time to reach the final state is the only remaining difference between the samples, and we suppose that it does not impact the droplet size considering the process as a whole. As a result, the addition of pure water to the first population of NEs gives a slight increase in the droplet diameter while also conserving the dispersion state of the population (PDI values). Consequently, the added water drops are also adsorbed by the reverse micellar phase and redistributed among the nano-droplets already present. This sequence is supported by the ability of different surfactant concentrations to accommodate the dispersed phase while conserving a good monodispersity (similarly to method A too).

### 3.5. Impact of the Nature of the Oil

**Figure 6:**
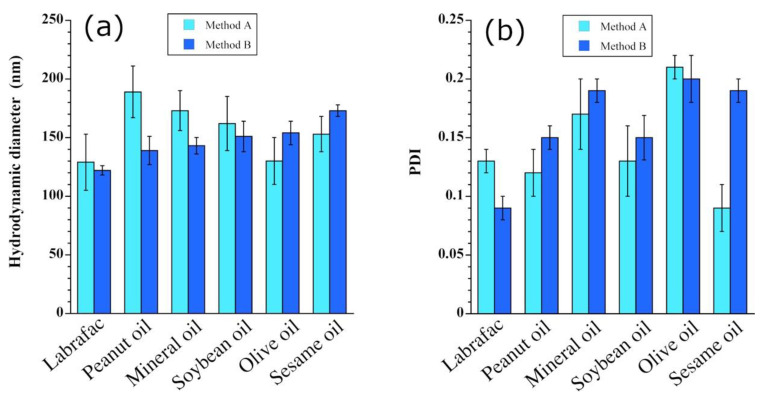
Influence of the nature of the oil on the spontaneous emulsification process and (**a**) the droplet size and (**b**) the corresponding PDI values. Experimental parameters: SOR 20%, WOR 10/90; the stirring rate and water addition rate for method B are 500 rpm and 0.4 mL/min, respectively; the surfactant is polyglycerol polyricinoleate (PGPR).

The last point investigated in this study was the effect of the nature of the oil on the process. A representative system was chosen that showed equivalency between method A and method B with Labrafac^®^ WL 1349, i.e., SOR 20%, WOR 10/90, stirring rate 500 rpm, and water addition rate for method B equal to 0.4 mL/min. The different oils were then tested for effects on droplet size (Figure 6a) and PDI values (Figure 6b). The results did not reveal any real breakthroughs between the different oils, except for a slight increase in the size and some fluctuations in the PDI as a function of the oils. The change in the size distribution probably reflects variations in the affinities between the surfactants and oil, and emphasizes the importance of this point in the emulsification process. Interestingly, the viscosities of these oils also vary [36], but viscosity shows no correlation with the results shown in Figure 6.

## 4. Conclusions

This study provided an in-depth investigation of the spontaneous emulsification that produces water-in-oil NEs. A series of spontaneous emulsification procedures were examined to investigate the changes in droplet size and polydispersity (PDI) in response to changes in: (i) the order of mixing, through a comparison between method A (oil added to water plus surfactant) and method B (water added to oil plus surfactant), (ii) the mixing rates, (iii) the amounts of surfactant, (iv) the types and mixtures of surfactants, (v) the amounts of dispersed phase, and (vi) the nature of the oil. The results provide evidence suggesting that the order of mixing of the compounds affects the emulsification mechanism, changing it from a turbulent phenomenon (method A) to a gradual emulsification, based on the presence of reverse micelles (method B). Further insights are presented as guides for the formulator regarding the choice of an appropriate system of formulation parameters (e.g., the volume fraction of the dispersed phase, the nature or concentration of the surfactant, or the nature of the oil) for the production of NEs that will meet the desired specifications. The methods evaluated in the present study could represent alternatives to high-energy methods and serve as a potential starting point for the design of complex vehicles for the encapsulation of hydrophilic therapeutic molecules.
